# *Mycobacterium bovi*s in Panama, 2013

**DOI:** 10.3201/eid2106.141821

**Published:** 2015-06

**Authors:** Fermín Acosta, Ekatherina Chernyaeva, Libardo Mendoza, Dilcia Sambrano, Ricardo Correa, Mikhail Rotkevich, Miroslava Tarté, Humberto Hernández, Bredio Velazco, Cecilia de Escobar, Jacobus H. de Waard, Amador Goodridge

**Affiliations:** Instituto de Investigaciones Científicas y Servicios de Alta Tecnología, Ciudad del Saber, Panama (F. Acosta, D. Sambrano, R. Correa, A. Goodridge);; Petersburg Institute of Phthisiopulmonology, St. Petersburg, Russia (E. Chernyaeva);; St. Petersburg State University, St. Petersburg (E. Chernyaeva, M. Rotkevich);; Inversiones para el Desarrollo de Coclé S.A., Anton, Panama (L. Mendoza);; Ministerio de Desarrollo Agropecuario, Tocumen, Panama (M. Tarté, H. Hernández, B. Velazco);; Instituto de Investigaciones Agropecuarias de Panamá, Ciudad del Saber (C. de Escobar);; Instituto de Biomedicina, Universidad Central de Venezuela, Caracas, Venezuela. (J.H. de Waard)

**Keywords:** zoonotic tuberculosis, bovine tuberculosis, biomarker, Mycobacterium bovis, whole-genome sequencing, single-nucleotide polymorphism, mycobacterial interspersed repetitive units, genotyping, tuberculosis and other mycobacteria, Panama

## Abstract

Panama remains free of zoonotic tuberculosis caused by *Mycobacterium bovis*. However, DNA fingerprinting of 7 *M. bovis* isolates from a 2013 bovine tuberculosis outbreak indicated minimal homology with strains previously circulating in Panama. *M. bovis* dispersion into Panama highlights the need for enhanced genotype testing to track zoonotic infections.

Zoonotic tuberculosis (TB) is a chronic infectious disease of humans caused by transmission of *Mycobacterium bovis* from cattle (*1*). *M. bovis* infection in humans occurs after direct contact with infected cattle, ingestion of unpasteurized dairy products or raw or undercooked meat, or (rarely) person-to-person transmission (*2*). Despite the low incidence of zoonotic tuberculosis in the Americas, accumulating evidence confirms that death rates from *M. bovis* pulmonary infection in specific groups and settings, including in the United States and Mexico, are substantial (*3*,*4*). The risk for death is twice as high for children and persons with HIV co-infection and extrapulmonary TB than for HIV-negative persons with TB (*3*). *M. bovis* infection in cattle (bovine TB) has a major effect on meat and live animal export trade and dairy industry development and expansion (*1*). Thus, bovine TB eradication plans across the Americas are based on the elimination of any cattle with a positive tuberculin skin test (TST) result (*5*). 

The most recently reported bovine TB outbreak in Panama occurred in 1997 in the western province of Bocas del Toro. The origin of this outbreak remains unclear. Since 2008 (after the slaughter of ≈7,000 cattle during the 1997 outbreak), Panama has not reported any bovine TB cases to the World Organisation for Animal Health (OIE) (Figure 1) (*6*). However, Panama has not received bovine TB-free accreditation. OIE data show that clinical bovine TB was continually reported from Colombia and Costa Rica during the same period (*6*). 

**Figure 1:**
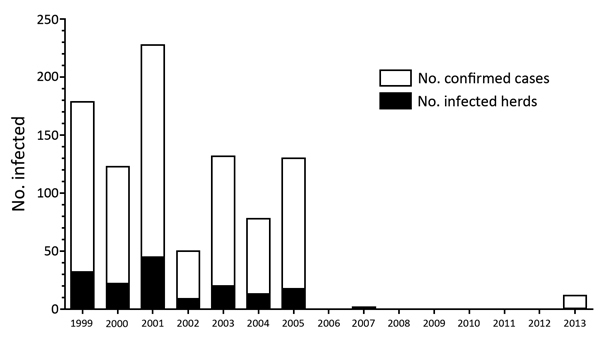
Bovine tuberculosis (TB) in Panama, 1999–2013. Data from the World Organisation for Animal Health (http://www.oie.int/animal-health-in-the-world/the-world-animal-health-information-system/the-oie-data-system/).

In August 2013, despite active surveillance at country borders and in-country animal health controls, a new bovine TB outbreak in Panama was reported to OIE (*6*). Neither the neighboring countries of Colombia and Costa Rica nor Panama have reported zoonotic tuberculosis to OIE in the past 20 years (*4*,*7*). Among these countries, only Costa Rica does not test *M. tuberculosis* complex isolates to identify *M. bovis*. In contrast, Guatemala continually reports cases of zoonotic TB (*6*). Yet, the genetic biodiversity of *M. bovis* in Central America remains unexplored. Comparisons of mycobacterial interspersed repetitive unit–variable-number tandem-repeat (MIRU-VNTR) and single-nucleotide polymorphism (SNP) analyses based on whole-genome sequencing have proven to be helpful for identifying TB outbreaks elsewhere (*8*,*9*). We characterized and genotyped *M. bovis* isolates that reemerged in Panama during the 2013 outbreak of bovine TB.

## The Study

In March 2013, as part of the Panamanian bovine TB control program, TSTs were administered to a dairy herd of 1,680 Jersey cows in Coclé Province, Panama. Animals with TST indurations >4 mm in diameter were considered positive. From animals with positive TST results, blood samples were collected for confirmation with an interferon-gamma release assay (BOVIGAM; Prionics, La Vista, NE, USA). Bovine TB was confirmed for 9 animals, 1 of which died naturally and 8 of which were sent to slaughter for postmortem examination and collection of samples for *M. bovis* culture. Epidemiologic investigation revealed that within the past 2 years, all 9 animals had been imported from Guatemala and Costa Rica according to controlled and legal commercial trade protocols. Visible lesions were found on all 8 slaughtered animals. Samples were obtained and sent for culture at the Laboratorio de Diagnóstico e Investigación Veterinaria de Salud Animal del Ministerio de Desarrollo Agropecuario.

**Figure 2:**
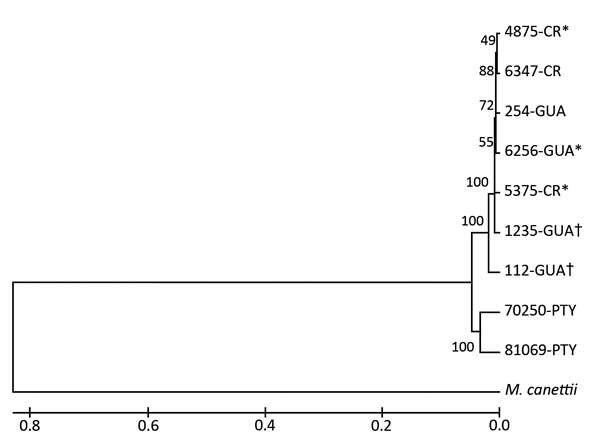
Phylogenic placement of the *Mycobacterium bovis* isolates. The *M. bovis* AF2122/97 sequence was used as a reference for single-nucleotide polymorphism analysis (GenBank accession no. NC_002945.3).The tree was derived from an unweighted pair-grouping method analysis algorithm by using DNA fragment sequence analysis. Numbers on branches represent bootstrap percentages from 500 replicates. *M. canettii* was used as the outgroup. Evolutionary analyses were performed by using MEGA6 software (http://www.megasoftware.net). Symbols indicate *M. bovis* isolates with identical genotypes according to mycobacterial interspersed repetitive units–variable number of tandem repeats. Scale bar indicates mean distances between strains according to base substitutions (%). CR, Costa Rica; GUA, Guatemala, PTY, Coclé, Panama.

Mycobacteria were isolated from 7 animals. Bacterial genomic DNA was extracted, and PCR identified the isolates as *M. bovis* (*10*). DNA from the *M. bovis* isolates was genotyped by 24-loci MIRU-VNTR (*11*). Whole-genome sequencing was performed by using the Illumina MiSeq platform (*12*), and sequencing reads were aligned on reference genome by using the bowtie2 program (http://bowtie-bio.sourceforge.net/bowtie2/index.shtml) and for further SNP calling and variant cell format file processing by using a combination of SAMtools (http://samtools.sourceforge.net) and VCFtools. *M. bovis* genome sequence NC_002945.3 was used as a reference for SNP/indel detection. Sequencing data for the *M. bovis* sequenced genomes were deposited in the National Center for Biotechnology Information Sequence Read Archive (accession no. PRJNA267480). SNP analysis revealed high homology among the 7 *M. bovis* isolates. However, the isolates were not directly related to 2 *M. bovis* isolates available from the 1997 bovine TB outbreak in Bocas del Toro (Figure 2).

Among the 7 isolates, a least four 24-loci MIRU-VNTR *M. bovis* genotypes were identified, including 5 distinct loci sequences (Table). These 4 distinct genotypes suggest that bovine TB may have been introduced by at least 4 infected animals. The *M. bovis* strains obtained from animals imported from Guatemala were clustered according to MIRU-VNTR genotypes (Table). Similarly, MIRU-VNTR genotypes for *M. bovis* isolates obtained from 3 animals imported from Costa Rica were more closely related. The 2 *M. bovis* isolates from the 1997 Bocas del Toro outbreak shared the same MIRU-VNTR genotypes with those from the recent outbreak. The molecular clock speed for this genotyping tool may account for the lack of differentiation in this last comparison, but we cannot rule out endemic spread of the same strain in this herd.

**Table:** The 24-loci MIRU-VNTR genotypes of *Mycobacterium bovis* isolates from outbreak of tuberculosis among cattle in Panama, 2013*

Isolate no.	MIRU							VNTR										MIRU							VNTR		
	04	26	40	10	16	31		42	43	ETR-A	47	52	53	QUB-11b	1955	QUB-26		02	23	39	20	24	27		46	48	49
BCG-4890†	2	2	5	4	2	2		4	3	2	4	4	3	4	3	4		1	3	3	3	3	2		5	1	2
254-GUA	2	2	5	4	2	2		2	3	2	1	7	3	4	5	4		2	2	3	4	3	2		3	1	2
1235-GUA	2	2	6	4	2	2		2	3	2	1	7	3	4	5	4		2	3	3	4	3	2		3	1	2
6256-GUA	2	2	6	4	2	2		2	3	2	1	7	3	4	5	4		2	3	3	4	3	2		3	1	2
112-GUA	2	2	6	4	2	2		2	3	2	1	7	3	4	5	4		2	3	3	4	3	2		3	1	2
4875-CR	2	2	6	4	2	2		2	3	2	1	7	3	5	5	4		2	3	3	4	3	2		3	1	2
5375-CR	2	2	6	4	2	2		2	3	2	1	7	3	5	5	4		2	3	3	4	3	2		3	1	2
6347-CR	2	2	3	4	2	2		2	3	2	4	7	3	5	5	4		2	5	3	3	3	2		3	1	2
81069-PTY	2	2	5	4	2	2		2	3	2	1	7	3	4	3	4		2	2	3	4	3	2		3	1	2
70250-PTY	2	2	6	4	2	2		2	3	2	1	7	3	5	3	4		2	3	3	4	3	2		3	1	2

*ETR, exact tandem repeats; MIRU-VNTR, mycobacterial interspersed repetitive units–variable number of tandem repeats; QUB, Queens University of Belfast.  †*Mycobacterium bovis* Bacillus Calmette Guérin American Type Culture Collection strain 4890.

SNP analysis revealed high genetic similarity among all 7 *M. bovis* isolates from the 2013 bovine TB outbreak. In contrast, we found no direct relation between *M. bovis* isolates from the 2013 and 1997 outbreaks. Because all 7 isolates were grouped in 2 branches, it is possible that they were derived from a common ancestor in Central America. A comparison of the SNPs from the 7 *M. bovis* isolates from the 2013 outbreak with 2 *M. bovis* isolates from the 1997 Bocas del Toro outbreak showed no close relationships. Adjacent countries have often reported *M. bovis* strains with highly similar genotypes, but geographically distant countries have not (*13*). 

We hypothesize that similar genotypes are being distributed in Central America because of augmented commercial trade between neighboring countries. Coincidentally, since January 2013, importation of cattle from Nicaragua, the United States, and Costa Rica into Panama for dairy and meat production has increased by ≈300%. Unfortunately, whole-genome sequencing data for *M. bovis* isolates from Central America are not available for validation of this hypothesis. Determination of the genetic structure of all *M. bovis* strains circulating in Central America will require further multicenter studies.

## Conclusions

The diagnosis of bovine TB in Panama requires urgent attention. Accumulated genetic changes in *M. bovis* isolates from 2 outbreaks occurred 15 years apart (*8*). Currently in Latin America, use of the TST remains the preferred method of identifying animals with bovine TB, despite its sensitivity range of 68% to 95% (*14*,*15*). In addition, several factors can lead to false-positive and false-negative TST results (e.g., purified protein derivative antigen quality and manipulation, skin induration interpretation, and injection dose protocols) (*15*). In contrast, interferon-gamma release assays are expensive and cost-prohibitive for rural farmers. For detection and quantification of the disease, novel and low-cost biomarker-based tests are needed; they would enable proper identification and disposal of diseased animals to prevent new outbreaks of zoonotic TB. After bovine TB is diagnosed, whole-genome sequencing and MIRU-VNTR can differentiate *M. bovis* lineages and identify patterns of bovine TB transmission across Central America.

Our study provides the baseline genotypes and sequences of *M. bovis* strains involved in the 2013 outbreak of bovine TB in Panama. These data could serve as a reference to determine future sources of zoonotic *M. bovis* infection and help track the movement of *M. bovis* strains between Central American countries. Together these strategies will reinforce international bovine TB control and eradication efforts.
